# Linking Sleep Disorders to Atrial Fibrillation: Pathways, Risks, and Treatment Implications

**DOI:** 10.3390/biology13100761

**Published:** 2024-09-25

**Authors:** Monica Ferreira, Mario Oliveira, Sergio Laranjo, Isabel Rocha

**Affiliations:** 1 Faculdade de Medicina and Centro Cardiovascular da Universidade de Lisboa-CCUL, Universidade de Lisboa, 1649-004 Lisbon, Portugal; ferreira_p_monica@hotmail.com (M.F.); mmartinsoliveira@gmail.com (M.O.); 2 Cardiology Department, Hospital de Santa Marta, Unidade Local de Saúde de S. José, 1150-199 Lisbon, Portugal; 3 Cardiology, Heart and Vessels Department, Hospital CUF Tejo, 1350-352 Lisboa, Portugal; sergiolaranjo@gmail.com; 4 CHRC, NOVA Medical School, Faculdade de Ciências Médicas, NMS, FCM, Universidade NOVA de Lisboa, 1169-056 Lisboa, Portugal

**Keywords:** sleep disorders, atrial fibrillation, obstructive sleep apnoea, cardiovascular risk

## Abstract

**Simple Summary:**

This review explores how common sleep disorders, such as insomnia and sleep apnoea, can affect heart health, particularly by increasing the risk of a heart condition called atrial fibrillation (AF). Sleep is vital for overall health, and disruptions in sleep can lead to significant health issues. Insomnia and sleep apnoea are widespread problems that affect millions of people worldwide. This paper examines how these sleep issues might cause changes in the body that lead to heart problems, such as inflammation and stress. The review also discusses how treating sleep disorders, especially with therapies such as continuous positive airway pressure for sleep apnoea, can help reduce the risk of developing AF and improve overall heart health. These conclusions are important because they suggest that addressing sleep disorders could be a key part of preventing and managing heart conditions, ultimately helping to improve people’s quality of life and reduce the burden of heart disease on society.

**Abstract:**

Sleep is a complex biobehavioural process essential for overall health, with various dimensions including duration, continuity, timing, and satisfaction. This study investigated the intricate relationships between common sleep disorders such as insomnia and obstructive sleep apnoea (OSA) and their impact on atrial fibrillation (AF), a prevalent arrhythmia with significant health implications. Using a comprehensive review of the current literature, this study examined the pathophysiological mechanisms linking sleep disorders to cardiovascular risks, focusing on autonomic nervous system disturbances, inflammation, and oxidative stress associated with OSA. These findings indicate that sleep disorders significantly elevate the risk of AF through mechanisms such as increased sympathetic activity and structural cardiac remodelling. Additionally, this study highlights the potential benefits of treating sleep disorders, particularly with continuous positive airway pressure (CPAP) therapy, in reducing AF recurrence and improving cardiovascular outcomes. This conclusion emphasises the importance of integrated therapeutic approaches that address both sleep disorders and AF to enhance patient outcomes and quality of life. Future research should explore these connections to develop more effective and holistic treatment strategies.

## 1. Introduction

Sleep is a complex biobehavioural process that is critical for maintaining overall health and wellbeing. It encompasses various dimensions, including duration, continuity, timing, rhythmicity, regularity, and satisfaction [1]. Accurate measurement of these dimensions is essential for understanding sleep health, with methods ranging from self-reported questionnaires to advanced technologies, such as polysomnography (PSG) and wrist actigraphy.

Among the most prevalent sleep disorders are insomnia and sleep-disordered breathing, which significantly affect a large portion of the population. Insomnia affects approximately 30% of individuals, with 5–10% meeting the clinical criteria for insomnia [2,3]. Sleep-disordered breathing, including obstructive sleep apnoea (OSA), affects 17% of the adult population and is frequently underdiagnosed, particularly in minority groups [4,5,6].

The pathophysiology of OSA is characterised by autonomic nervous system disturbances, intermittent hypoxia, and intrathoracic pressure changes, which contribute to systemic inflammation and oxidative stress [7,8]. These factors collectively elevate cardiovascular risk and link sleep disorders to conditions such as atrial fibrillation (AF).

AF is a common and serious arrhythmia, with significant morbidity, mortality, and increased prevalence. Sleep disorders are now recognised as independent risk factors for AF and their interplay with cardiovascular health is an emerging area of research. Studies suggest that mechanisms such as increased sympathetic activity, inflammatory responses, and oxidative stress are pivotal in the development and progression of AF in patients with sleep disorders [9,10,11].

While significant progress has been made in understanding how OSA contributes to AF, the pathophysiological mechanisms by which other sleep disorders, such as insomnia and restless leg syndrome, influence AF development and progression remain largely unexplored.

There exists an unmet need for a multidisciplinary approach that integrates sleep disorder treatment into the management plan for AF patients, potentially improving outcomes and quality of life for this patient population.

This review aimed to elucidate the complex relationships between sleep disorders, particularly OSA, insomnia, and AF. By exploring the underlying pathophysiological mechanisms and the impact of treating sleep disorders on AF outcomes, we aimed to provide a comprehensive understanding that can inform clinical practice and guide future research. In order to achieve this, we conducted a comprehensive literature search to examine the link between sleep disorders and AF. We systematically searched peer-reviewed databases, including PubMed and Google Scholar, using keywords such as ‘sleep disorders’, ‘insomnia’, ‘obstructive sleep apnoea’, and ‘atrial fibrillation’. We focused on studies exploring the pathophysiological mechanisms connecting sleep disorders to AF, such as autonomic dysfunction, inflammation, and oxidative stress as well as clinical management strategies. We included experimental studies, clinical trials, cohort studies and meta-analyses published in English over the past 20 years. The selected studies were synthesised into key thematic sections to clarify how sleep disorders contribute to AF development and the potential benefits of treating these disorders to improve AF outcomes. Thus, the significance of addressing sleep disorders in the management of AF has been highlighted, emphasising the potential for improving patient outcomes through integrated therapeutic approaches.

## 2. Most Common Sleep Disorders

### 2.1. Insomnia

Insomnia is the most common sleep disorder and complaint affecting approximately 30% of the population, with 5–10% meeting the clinical criteria for insomnia disorder [2,12]. The American Psychiatric Association defines insomnia as experiencing symptoms at least three nights per week for a duration of at least three months, despite having adequate opportunities for sleep. These symptoms must significantly distress or impair daytime functioning and cannot be attributed to substance abuse or medication [13]. Diagnosis of insomnia can be achieved through a clinical interview by a trained professional without necessarily requiring objective evidence from polysomnography (PSG). Symptoms of insomnia can also be assessed using standardised tools such as the Insomnia Severity Index (ISI) [14] or by evaluating specific symptoms such as difficulty in initiating sleep. Objective assessments of sleep continuity, such as sleep latency (SL), wakefulness after sleep onset (WASO), and sleep efficiency (SE; calculated as the proportion of time spent asleep while in bed), can be conducted using actigraphy or PSG [15].

### 2.2. Sleep-Disordered Breathing 

Sleep-disordered breathing encompasses a variety of disorders including obstructive sleep apnoea (OSA), central sleep apnoea (CSA), and sleep-related hypoventilation disorders. These conditions affect approximately 20–30% of adult men and 15% of adult women [4]. The primary mechanisms leading to respiratory disruption during sleep include upper airway obstruction, respiratory control dysregulation, and hypoventilation. Among them, OSA and CSA were the most prominent. The severity of these disorders is assessed based on the apnoea-hypopnoea index (AHI), which counts the number of respiratory events per hour of sleep and the extent of oxygen desaturation [16,17]. Sleep-disordered breathing was categorised as mild with an AHI of 5–15 events per hour, moderate with an AHI of 15–30 events per hour, and severe with an AHI greater than 30 events per hour. OSA is the most prevalent form of sleep-disordered breathing in the general population and is characterised by recurrent partial (hypopnoea) or complete (apnoea) collapse of the upper airway during sleep, resulting in reduced or interrupted airflow despite ongoing respiratory effort [18].

### 2.3. Obstructive Sleep Apnoea (OSA)

**Figure A1:**
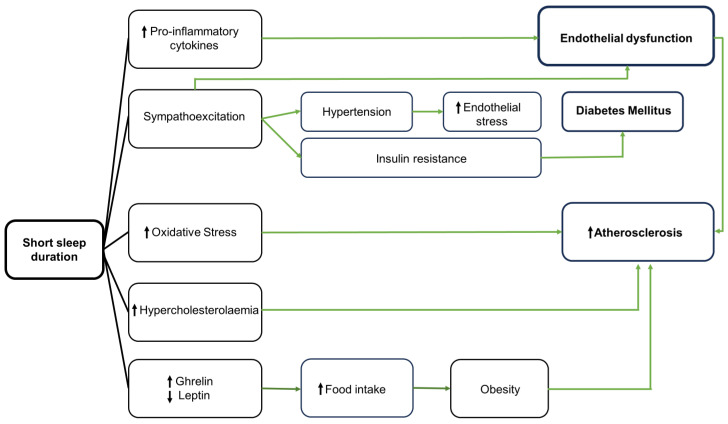
Legend: Mechanistic pathways connecting reduced sleep duration with elevated cardiovascular disease risk. Insufficient sleep triggers various biological processes, including dysregulation of the autonomic nervous system, heightened inflammation, increased oxidative stress and altered lipid metabolism.

Obstructive sleep apnoea (OSA) affects approximately 17% of the adult population and its prevalence is steadily increasing. However, it remains underdiagnosed, particularly in the minority population. It is estimated that 1 in 15 individuals experience at least a moderate form of OSA [5,6]. The pathophysiology of OSA involves disturbances in the autonomic nervous system, intermittent hypoxia, and intrathoracic pressure alterations [19,20] (Figure A1 in Appendix A). These intermittent bouts of hypoxia or reduced oxygen levels occur as airflow is obstructed despite continuous thoracic and abdominal effort [7]. The resultant intrathoracic pressure changes, combined with increased CO_2_ and decreased oxygen levels, contribute to the systemic inflammation and oxidative stress. Studies suggest that the sympathetic activation changes seen during sleep in OSA patients persist during wakefulness [9,10]. Microneurographic recordings of sympathetic nerve activity in the peroneal nerve have demonstrated that the rate of sympathetic bursts doubles and that their amplitude increases in individuals with OSA. During OSA episodes, diastolic function is impaired and atrial and aortic enlargement occurs. Thin-walled atria are particularly susceptible to the intrathoracic pressure swings caused by OSA. These pressure variations lead to a shift in the intraventricular septum, resulting in a decreased cardiac output. Additionally, low oxygen levels during episodes increase the mean pulmonary arterial pressures [20,21,22,23]. Other studies have linked OSA with systemic inflammation and prothrombotic effects, both of which may elevate cardiovascular or atherogenic risk [8]. Furthermore, oxidative stress occurs as intermittent bouts of low oxygen levels cause oxidation of serum proteins and lipids. Endothelial dysfunction, insulin resistance, and dyslipidaemia have also been observed [21]. Collectively, these factors contribute to atherogenesis and increase the cardiovascular risk. In particular, OSA is increasingly recognised as a significant risk factor for the development and progression of AF. In patients with AF, the prevalence of OSA is notably high, with some studies indicating that up to 50% of AF patients may have undiagnosed OSA [24,25]. The severity of OSA, often quantified by the apnoea–hypopnea index (AHI), is directly correlated with an increased risk of developing AF and a higher likelihood of AF recurrence following treatments like cardioversion and ablation [24,25]. Specifically, patients with severe OSA are at an elevated risk due to the recurrent episodes of hypoxia and hypercapnia, which exacerbate atrial electrical instability and autonomic imbalance, making the atria more susceptible to arrhythmias [24,25].

## 3. Pathophysiologic Mechanisms Linking Short Sleep Duration and Cardiovascular Diseases 

### 3.1. Inflammation 

Inflammation is a significant mechanism contributing to an increased risk of cardiovascular disease in individuals with short sleep durations [26]. Studies have shown that even after just one night of partial sleep deprivation (restricted to 4 h), there is an increase in mRNA levels and the production of pro-inflammatory cytokines, such as IL-6 and tumour necrosis factor (TNF) [27]. Research on the effects of acute total sleep deprivation in real-world settings found elevated levels of type II interferon (IFNγ), while the levels of IL-2, IL-10, and TNF remained unchanged [28]. Acute sleep deprivation disrupts the circadian pattern of IL-6 secretion, resulting in excessive secretion during the day and under-secretion at night. High levels of IL-6 have been hypothesised to contribute to daytime sleepiness and fatigue [29]. Additionally, C-reactive protein (CRP), a well-known predictor of cardiovascular events [28,30], has been reported to be elevated in individuals experiencing 88 h of sleep deprivation or partial sleep deprivation (4 h per night for 10 days) [31].

### 3.2. Oxidative Stress and Endothelial Dysfunction

Oxidative stress represents another potential mechanism that contributes to the increased risk of cardiovascular diseases associated with short sleep duration. Reactive oxygen species (ROS) influence myocardial contractility, trigger arrhythmias, and induce cardiac remodelling through hypertrophic signalling, apoptosis, and necrosis [32]. ROS play crucial roles in various vascular cell functions, including endothelial and smooth muscle cell growth, proliferation, migration, angiogenesis, apoptosis, vascular tone, and host defence. However, excessive ROS levels can lead to vascular diseases through direct and irreversible oxidative damage [32]. Short-term sleep deprivation has been linked to elevated levels of myeloperoxidase, an enzyme involved in forming oxidising agents that can convert LDL cholesterol into an atherogenic form [33]. Sleep deprivation has also been associated with increased levels of insulin-like growth factor I (IGF1), which is involved in releasing oxidant agents from activated neutrophils [34]. In the Chicago Area Sleep Study [35], which enrolled individuals at low risk for OSA and objectively assessed sleep duration, no connection was found between sleep duration and prothrombotic markers.

### 3.3. Autonomic Nervous System 

The ANS has been investigated as a potential mechanism for the increased risk of cardiovascular disease associated with short sleep. There is strong evidence of increased sympathetic activity following partial or total acute sleep deprivation [36] or sleep fragmentation [37]. A reduction in HRV and a shift in sympathovagal balance towards sympathetic predominance have been reported after overnight total sleep deprivation [38]. Acute sleep deprivation results in increased sympathetic modulation directed towards the heart, as evidenced by an increase in the low-frequency component and the low-frequency/high-frequency ratio of HRV, reduced vagal control, and blunted responses to orthostatic challenges evaluated by head-up tilt testing [38]. Furthermore, increased low-frequency components of blood pressure variability and decreased BRS have been observed after total sleep deprivation [38]. As a result, acute sleep deprivation has significant effects on haemodynamic and autonomic parameters. In fact, efferent activity in the vagus nerve leads to the release of ACh in the organs of the reticuloendothelial system, and ACh interacts with receptors on tissue macrophages, inhibiting the release of IL-1, high-mobility group protein B1, TNF, and other cytokines [39]. Consequently, the autonomic imbalance observed after acute total or partial chronic sleep deprivation may promote a pro-inflammatory state [39].

### 3.4. Circadian Rhythms 

Circadian rhythms are approximately 24 h variations in physiological and mental processes, governed by an endogenous biological clock that responds to external signals such as light. The human circadian system has evolved to synchronise with the Earth’s light–dark cycle. As a result, certain physiological functions and behaviours typically occur during daytime hours (e.g., waking, eating, and physical activity), while others typically occur during night-time hours (e.g., sleeping and fasting). Circadian misalignment can contribute to various mental and physical disorders, particularly cardiovascular risk [40]. This misalignment may explain the long-standing clinical observation of a heightened risk of adverse cardiovascular events during the morning hours [41]. Disturbances in circadian rhythms can lead to circadian misalignment, which disrupts the temporal harmony that usually exists between the endogenous circadian system and the timing of behaviours such as sleeping/waking and eating/fasting [41]. 

### 3.5. Chronotype

A chronotype is a concept that describes an individual’s temporal organisation or diurnal preference, influenced by genetic, demographic (e.g., age, sex), and environmental factors (e.g., light exposure). Chronotype can be assessed through questionnaires [42,43] about one’s preferred sleep–wake times and subjective times of peak performance and alertness, or quantified by evaluating sleep timing as a behavioural manifestation of the internal circadian timekeeping system [44]. Having a relatively later chronotype (i.e., an evening vs. morning preference) is associated with several potential contributors to cardiovascular disease (CVD) risk, including metabolic dysfunction [45], poor health behaviours such as smoking [46], unhealthy diets [47], low physical activity, and high alcohol consumption [48]. Conversely, having an evening (vs. morning) chronotype was associated with higher odds of HTA [49]. Further studies are needed to determine whether chronotype is associated with the development and progression of CVD. External light exposure is the most potent cue that synchronises an individual’s biological timing to the solar 24 h day, and as such, it is a modifiable determinant of chronotype [50].

## 4. Sleep and Atrial Fibrillation 

**Figure A2:**
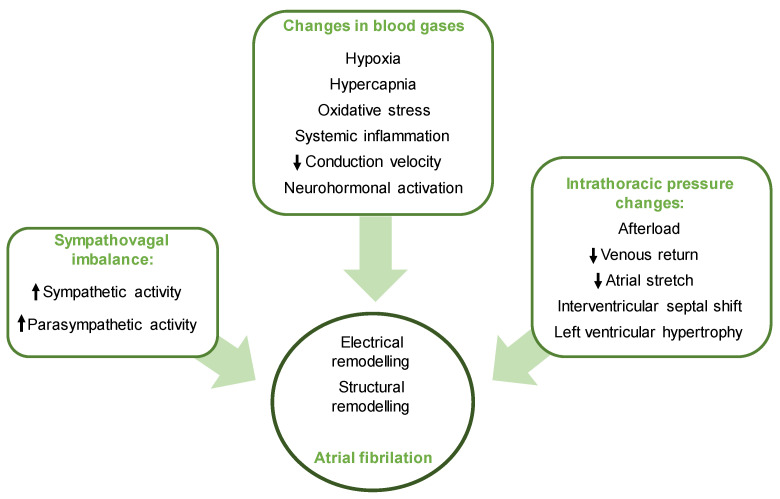
Legend. Various potential pathways through which sleep disturbances might contribute to the development of atrial fibrillation. Sleep disorders can influence atrial fibrillation through mechanisms such as autonomic nervous system imbalance, increased inflammation, oxidative stress, and changes in cardiovascular haemodynamics.

The connection between sleep and heart health is well established. Sleep disorders are considered to be independent risk factors for several cardiovascular diseases, including AF. There is considerable debate regarding the results of studies investigating the association between sleep duration and AF incidence. Sleep deprivation has been linked to alterations in ECG parameters including signs of AF [51]. Furthermore, sleep deprivation is associated with P-wave dispersion, QT dispersion, and P-wave duration, which are also predictors of AF [51,52]. Short sleep duration negatively affects endocrine, immunological, and metabolic systems [47,53] (Figure A2). Additionally, sleep deprivation in healthy adults is associated with a reduction in the left atrial early diastolic strain rate [54]. In the MESA (Multi-Ethnic Study of Atherosclerosis) Sleep Study, a longer duration of slow-wave sleep (SWS) was associated with lower odds of AF [55]. The association between SWS (state with the highest parasympathetic activity) duration and AF underscores the importance of sleep duration for sleep health. Generally, sleep is associated with high parasympathetic and low sympathetic activities, which might be related to AF occurrence [51]. In a study by Ayas et al. [56], both short and long sleep durations were associated with a higher risk of AF in different studies, suggesting a U-shaped association between sleep duration and AF.

### 4.1. Sleep Disordered Breathing and AF

Most sleep apnoea events in AF patients are obstructive respiratory events, characterised by repetitive partial (obstructive hypopnoea) or complete (obstructive apnoea) collapse of the upper airway during sleep [57]. Central apnoeas primarily involve alternating hyperventilation and hypoventilation, reflecting intermittent hypoxaemia and hypo- and hypercapnia. Obstructive respiratory events are also linked to intrathoracic pressure fluctuations (down to −60 mmHg) resulting from inefficient inspiration against the obstructed upper airways. Both the cardiac haemodynamic and transmural pressure gradients change significantly during obstructive respiratory events. While hypoxaemia accumulates, the event is terminated by arousal activation driven by the combined sympathovagal activation of the autonomic nervous system [58]. A study in rats with chronic sleep-disordered breathing (SDB) simulation, induced by either upper airway obstruction or applying negative airway pressure via a customised facial mask for up to four weeks, resulted in connexin dysregulation and increased atrial fibrosis formation. This was associated with structural remodelling, conduction abnormalities, and increased AF inducibility [59]. Correspondingly, patients with long-term SDB show marked atrial structural changes and conduction abnormalities in the atria, without any changes in atrial refractoriness, forming a substrate for AF vulnerability [60]. This was predominantly observed in patients with paroxysmal AF, and SDB severity was associated with advanced remodelling in patients with persistent AF. This stress is a critical contributor of SDB to AF substrate progression [60]. Atrial structural remodelling may contribute to the maintenance of AF [61,62,63,64]. Clinical observations suggest that nocturnal AF paroxysms are often associated with individual respiratory obstructive events [64]. In the Variability of Sleep Apnoea Severity and Risk of Atrial Fibrillation (VARIOSA-AF) study, patients with implanted pacemakers showed considerable night-to-night variability in sleep apnoea severity, and severe sleep apnoea nights conferred a 1.7-fold increased risk of having at least five minutes of AF during the same day compared with the best sleep nights [65]. This indicates that acute transient arrhythmogenic changes during apnoea may further contribute to the development of AF. In a pig model of obstructive respiratory events, the application of negative tracheal pressure (−50 mbar) during tracheal occlusion reproducibly and reversibly shortened the atrial refractory period and enhanced AF inducibility [58]. In AF patients, SDB is associated with an increased incidence of extrapulmonary vein triggers [64,66], which may be explained by increased calcium/calmodulin-dependent protein kinase II (CaMKII)-dependent phosphorylation of the cardiac voltage-gated sodium channel (NaV1.5) [67].

Although SDB, such as sleep apnoea, has often been studied in relation to AF, other sleep disorders also have important implications. These include insomnia, restless legs syndrome, and circadian rhythm disorder.

### 4.2. Insomnia and AF

Recent research suggests that insomnia may independently contribute to the risk of AF. A large cohort study of more than 14,000 individuals found that those who reported frequent insomnia symptoms had a 29% higher risk of developing new-onset AF over a median follow-up period of nearly a decade, even after adjusting for sleep duration and SDB [68]. Although the mechanisms underlying this association are not entirely clear, insomnia-induced hyperarousal, a state of heightened physiological and psychological activation, may play a key role. Hyperarousal can lead to increased SNS activity and decreased PNS activity, both of which may contribute to the pathogenesis of AF. 

### 4.3. Circadian Rhythm Disorders

Circadian rhythm disorders, in which the individual sleep–wake cycle and light–dark cycle of the environment do not coincide, could also be associated with AF. Shift work, which disrupts normal sleep–wake rhythms, has been linked to an increased risk of AF [69]. This may be due to a combination of circadian dysregulation, sleep deprivation, and other lifestyle factors that are associated with shift work. In addition, the molecular mechanisms underlying circadian rhythms may directly influence cardiac electrophysiology. Disruptions in circadian rhythm genes such as “Clock” and “Bmal1” have been shown to influence action potential duration and promote arrhythmogenesis in animal models [70].

The direct association between these sleep disorders and AF highlights the importance of comprehensive sleep studies in patients with AF. Understanding the contribution of these sleep disorders to AF could also open new therapeutic avenues. For example, improving sleep hygiene, treating insomnia with cognitive behavioural therapy, treating RLS with appropriate medications, and minimising circadian disruption could potentially help prevent AF and reduce its severity in already diagnosed patients. However, more research is needed to confirm these potential strategies and further explore the complex interplay between sleep disorders and AF. 

### 4.4. Sleep Quality

Sleep quality is an essential aspect of overall sleep health. While sleep duration is the main focus in the context of AF, there is a growing body of research on the possible role of sleep quality in the development and progression of this condition. Sleep quality can be quantified using several parameters, including SE, sleep latency, frequency of awakenings, and depth of sleep, as well as the subjective feeling of rest and recovery after sleep. Various studies have linked poor sleep quality, characterised by difficulty falling asleep, frequent night-time awakenings, or non-restorative sleep, to an increased risk of cardiovascular disease, including AF. In the I-STOP-AFIB (Individualised Studies of Triggers of Paroxysmal Atrial Fibrillation), trial patients reported sleep quality on a daily basis. Their poor sleep was associated with an immediately heightened risk for self-reported AF episodes, and a dose-response relationship existed such that progressively worse sleep was associated with longer episodes of AF the next day [71].

The association between sleep quality and AF is supported by several mechanisms. Sleep fragmentation, a feature of poor sleep quality characterised by frequent arousals and sleep interruptions, is known to lead to activation of the SNS, a reduction in parasympathetic activity, an increase in HR, and an increase in BP. All of these changes could potentially favour the development of AF [70]. In addition, fragmented sleep can trigger inflammatory processes and oxidative stress, both of which are involved in the development of AF [72,73,74]. 

The quality of sleep is not only about interruptions but also about the depth of sleep. Slow-wave sleep (SWS), the deepest phase of sleep (not rapid eye movement (REM) sleep), is associated with parasympathetic dominance and cardiovascular recovery. A decrease in SWS has been associated with an increased risk of HTA, a recognised risk factor for AF [75]. In one study, AF was associated with impaired sleep quality, as indicated by a lower quantity of SWS [55]. Therefore, the possible role of reduced SWS in the pathogenesis of AF should be further investigated.

In addition, the subjective feeling of non-restorative sleep, even with sufficient sleep duration, could play a role in AF. Individuals who consistently report non-restorative sleep often experience increased daytime sleepiness, decreased energy, and impaired concentration [76]. It has been postulated that non-restorative sleep could disrupt vital recovery processes that occur during sleep, including the breakdown of metabolites in the brain and the regulation of inflammatory processes [76]. These disruptions could potentially impact cardiovascular health and predispose to AF [76].

### 4.5. Impact of Treating Sleep Disorders in AF

As previously stated, sleep disorders, particularly OSA, significantly affect AF incidence and progression [77,78,79,80]. There is growing evidence that the successful treatment of sleep disorders can potentially change the outcomes of AF. Continuous positive airway pressure (CPAP) is the first-choice treatment for OSA. It ensures upper airway patency and prevents the development of apnoeas and hypopneas. The use of CPAP is associated with several cardiovascular benefits, including lowering BP and improving left ventricular function [81]. Some studies mention that the use of CPAP in patients with OSA and AF significantly reduces the risk of AF catheter ablation recurrence [81,82]. The impact of CPAP therapy extends beyond reducing AF recurrence. A recent study [61] showed that treatment of OSA with CPAP resulted in a reduction in left atrial size, suggesting that CPAP may reverse the atrial remodelling associated with OSA, thereby attenuating one of the factors contributing to AF.

CPAP therapy also improves quality of life and reduces daytime sleepiness in patients with AF and OSA. These improvements may indirectly impact AF management by improving patient adherence to AF and promoting healthier lifestyle habits [83]. In addition, CPAP therapy can potentially influence stroke risk in patients with AF. OSA was independently associated with a higher risk of stroke in patients with AF. Gupta and Shukla [84] showed that effective treatment of OSA with CPAP resulted in a lower incidence of stroke in AF patients. Despite growing evidence in favour of CPAP therapy, patient adherence to CPAP therapy remains suboptimal. Factors contributing to non-adherence include discomfort with the machine, stuffy nose, dry mouth, and noise. It is important to address these barriers to optimise the cardiovascular benefits of CPAP therapy [85]. 

One alternative is the use of mandibular advancement devices (MADs), which are oral appliances that reposition the lower jaw forward, thereby increasing airway space and reducing the likelihood of airway collapse during sleep. MADs are particularly effective in patients with mild to moderate OSA and those who cannot tolerate CPAP [86,87].

Positional therapy is another alternative, which involves strategies to prevent patients from sleeping in positions that exacerbate OSA, such as the supine position. Devices such as specialised pillows or belts can help maintain a side-sleeping position, reducing the severity of OSA in some patients [88,89,90].

Weight loss, particularly in overweight and obese individuals, is a critical component of managing OSA. Weight reduction can significantly decrease the severity of OSA and, consequently, the burden of AF. Behavioural changes, increased physical activity, and dietary modifications are essential elements of a comprehensive treatment plan [91,92].

Surgical interventions, such as uvulopalatopharyngoplasty (UPPP) or maxillomandibular advancement (MMA), may be considered in severe cases where other treatments have failed [93]. These surgeries aim to reduce airway obstruction by removing or repositioning tissues in the upper airway.

For patients with morbid obesity, bariatric surgery can lead to substantial weight loss and improvement in OSA symptoms, which may reduce the risk of AF [94,95]. Additionally, lifestyle changes such as alcohol abstinence and smoking cessation have beneficial effects on both OSA and AF outcomes [96,97].

Regarding the usage of drugs, the effectiveness of antiarrhythmic drugs (AADs) in managing AF is significantly influenced by the presence and severity of OSA. Research has shown that patients with severe OSA exhibit a reduced response to AAD therapy compared to those with mild or no OSA. For example, a study by Monahan and colleagues [98] demonstrated that severe OSA was associated with a markedly lower success rate of rhythm control when using AADs. Specifically, only 39% of patients with severe OSA responded to AAD therapy, compared to 70% of those with mild or moderate OSA [98]. The underlying reason for this reduced efficacy lies in the pathophysiological mechanisms associated with OSA, such as recurrent hypoxia and increased sympathetic nervous system activity, which contribute to atrial remodelling and autonomic dysfunction. These factors collectively reduce the stability of the atrial myocardium, making it less responsive to the stabilising effects of AADs. Therefore, when initiating AAD therapy in AF patients, it is crucial to assess and manage OSA, as untreated OSA can significantly diminish the effectiveness of these drugs [98,99].

Beta-blockers are commonly prescribed in the management of AF to control heart rate and reduce the frequency of arrhythmic episodes. However, recent evidence suggests that beta-blockers may not be as safe or effective in patients with OSA as previously believed. A large population-based cohort study by Chen and colleagues [100] found that beta-blocker use in patients with OSA was associated with an increased risk of cardiovascular disease (CVD) outcomes and a trend toward increased all-cause mortality.

This increased risk may be attributed to the complex interactions between beta-blockers and the autonomic nervous system in the context of OSA. OSA is characterised by heightened sympathetic activity and intermittent hypoxia, conditions that beta-blockers could potentially exacerbate, leading to adverse cardiovascular outcomes. Thus, while beta-blockers remain a standard treatment for AF, their use in patients with OSA should be approached with caution. Close monitoring is essential, and alternative therapies should be considered, particularly for patients with severe OSA who may be at higher risk of adverse effects [25].

## 5. Conclusions and Future Perspectives

In conclusion, the interplay between sleep disorders and atrial fibrillation (AF) is both multifaceted and significant. Sleep disorders, such as insomnia and obstructive sleep apnoea (OSA), share common risk factors for AF, including obesity, hypertension, and aging, and contribute to the progression of AF through mechanisms such as autonomic nervous system activation, increased inflammatory response, and oxidative stress.

Obstructive sleep apnoea, in particular, has been extensively linked to AF owing to its effects on autonomic regulation and cardiovascular remodelling. Repeated episodes of hypoxia coupled with fluctuations in intrathoracic pressure during sleep create a conducive environment for AF development. These physiological changes persist even during wakefulness, highlighting the chronic impact of sleep apnoea on cardiovascular health.

Insomnia, another prevalent sleep disorder, independently increases the risk of AF, possibly through mechanisms involving hyperarousal and increased sympathetic activity. Furthermore, circadian rhythm disruption and poor sleep quality contribute to cardiovascular risks, including AF, by disrupting normal autonomic function and promoting inflammatory processes.

Addressing sleep disorders through targeted interventions such as continuous positive airway pressure (CPAP) therapy for OSA has shown promising results in reducing AF recurrence and improving overall cardiovascular outcomes. The effective treatment of sleep disorders not only enhances sleep quality but also mitigates adverse effects on cardiovascular health, offering a holistic approach to managing AF.

Future research should continue to explore the complex mechanisms linking sleep disorders and AF, with an emphasis on developing comprehensive treatment strategies that simultaneously address both conditions. Understanding these relationships will enable healthcare providers to design more effective interventions, ultimately improving patients’ outcomes and quality of life.

## Data Availability

Data sharing is not applicable to this article.
